# Using MARSIS signal attenuation to assess the presence of South Polar Layered Deposit subglacial brines

**DOI:** 10.1038/s41467-022-33389-4

**Published:** 2022-09-28

**Authors:** Sebastian E. Lauro, Elena Pettinelli, Graziella Caprarelli, Jamaledin Baniamerian, Elisabetta Mattei, Barbara Cosciotti, David E. Stillman, Katherine M. Primm, Francesco Soldovieri, Roberto Orosei

**Affiliations:** 1 Dipartimento di Matematica e Fisica, Università degli studi Roma Tre, Rome, Italy; 2 Centre for Astrophysics, Institute for Advanced Engineering and Space Sciences, University of Southern Queensland, Toowomba, QLD Australia; 3 Department of Space Studies, Southwest Research Institute, Boulder, CO USA; 4 Planetary Science Institute, Tucson, AZ USA; 5 Istituto per il Rilevamento Elettromagnetico dell’Ambiente, Consiglio Nazionale delle Ricerche, Naples, Italy; 6 Istituto di Radioastronomia (IRA), Istituto Nazionale di Astrofisica (INAF), Bologna, Italy

**Keywords:** Inner planets, Cryospheric science

## Abstract

Knowledge of the physical and thermal properties of the South Polar Layer Deposits (SPLD) is key to constrain the source of bright basal reflections at Ultimi Scopuli detected by the MARSIS (Mars Advanced Radar for Subsurface and Ionosphere Sounding) radar sounder. Here we present a detailed analysis of attenuation, based on data acquired by MARSIS at 3, 4, and 5 MHz. We show that attenuation is frequency dependent, and that its behavior is consistent throughout the entire region. This suggests that the SPLD are compositionally homogeneous at Ultimi Scopuli, and our results are consistent with dust contents of 5 to 12%. Using these values as input, and plausible estimates of surface temperature and heat flux, we inferred basal temperatures around 200 K: these are consistent with perchlorate brines within liquid vein networks as the source of the reflections. Furthermore, extrapolation of the attenuation to higher frequencies explains why SHARAD (Shallow Radar) has thus far not detected basal reflections within the SPLD at Ultimi Scopuli.

## Abstract (web summary)

MARSIS attenuation and thermal data confirm that liquid brines are the most plausible source for the bright reflections at the base of the South Polar Layered Deposits. Such results also justify why SHARAD does not penetrate to the base of the ice.

## Introduction

Bright basal reflections detected by the radar sounder MARSIS (Mars Advanced Radar for Subsurface and Ionosphere Sounding) at Ultimi Scopuli^1,2^ sparked a heated debate on the nature and thermal properties of the materials within the South Polar Layers Deposits (SPLD) and at the base of the ice. Interpretations for the source of the reflections vary, with some authors proposing basal liquid brines^1–3^, and others suggesting solid conductive materials, such as saline ice^4^, hydrated clays^5^ or Fe-rich basalt with high contents of ilmenite^6^. A recent paper has discussed dielectric theory, extensive literature data and new experimental results, showing that no lines of evidence support saline ices or hydrated clays as the source of the bright basal reflections^3^, however the critical question of how liquid brines could form and persist at the base of the SPLD remains. Critics of the brine interpretation argue that, unless an anomalously high geothermal gradient is assumed^7^, basal temperatures should not exceed $180\,{{{{{\rm{K}}}}}}$, well below the eutectic temperatures of the salts likely to be found in the region (perchlorates and chlorides). Related to the question of the basal temperatures is the composition of the SPLD, which affects the thermal properties of the deposits and determines the temperature gradients within the deposits themselves, thus influencing by how much the temperature increases from the surface to the base of the SPLD. It is generally accepted that the SPLD are primarily an admixture of water ice and dust. Radar data from MARSIS acquired over the south polar cap have been interpreted to indicate dust contents of 10%^8^, while interpretation of gravity data calculated from observations of Doppler tracking of the Mars Reconnaissance Orbiter (MRO) spacecraft suggested a dust content of ~15%^9^ and a density of the deposits of $1220\,{{{{{\rm{kg}}}}}}/{{{{{{\rm{m}}}}}}}^{3}$. Lithospheric flexure models of ice cap loading have resulted in a best fit density value of $1271\,{{{{{\rm{kg}}}}}}/{{{{{{\rm{m}}}}}}}^{3}$, from which relative proportions of 14–28% dust content were obtained^10^. Using radar data to constrain the results of an elastic loading model of the lithosphere returned a best fit density of $1220\,{{{{{\rm{kg}}}}}}/{{{{{{\rm{m}}}}}}}^{3}$ which, for dust density values ranging from 2200 to 3400 kg/m^3^, corresponds to dust contents between 9 and 18% (if no CO_2_ is considered)^11^.

The presence of liquid salty water at a depth of ~1.5 km below the surface of the SPLD in Ultimi Scopuli was first inferred from MARSIS data^1^ using an inversion method^12^, from which two distinct distributions of apparent permittivity values were retrieved: a high value distribution, characterizing the bright area, interpreted as evidence of basal brines; and a low value distribution, detected for the surrounding areas, which is typically attributed to dry and frozen rocks or soil^1^. Following this study, other bright basal reflections were detected in the vicinity of the site: Lauro et al.^2^ applied a signal processing technique commonly used in terrestrial Radar Echo Sounding (RES) investigations to discriminate between wet and dry subglacial basal conditions, which reinforced the interpretation that the bright basal reflections were due to the presence of basal brines. Working independently, Carrer and Bruzzone^13^ further showed that a novel approach, based on the relationship between radar surface and subsurface reflections, supports the interpretation of subglacial brines in Ultimi Scopuli.

The reporting of new bright areas in other regions of the south polar cap^14^, including in locations where the SPLD are thinner, added a new puzzling aspect to the debate, while another recently published paper proposed that volcanic rocks covered by a 1.5 km thick ice sheet could produce bright reflections consistent with those measured at Ultimi Scopuli^6^. It should be noted however, that these two interpretations rely on data processed on board the spacecraft, which are averages of groups of 100–300 raw echoes after compensating for the vertical motion of the spacecraft^15^. This data acquisition process is different than that utilized by Orosei et al.^1^ and Lauro et al.^2^, who acquired raw data targeting the specific area of interest^16^. On board processed observations at Ultimi Scopuli are at least an order of magnitude more numerous than raw data, but are spaced several kms along the ground track, and their ratio of surface to subsurface echo power is potentially affected by small errors in the compensation of the vertical motion of the spacecraft. Raw echoes, instead, are less than 100 m apart along the ground track and preserve the full information on echoes acquired from the entire area illuminated by the radar (Supplementary Fig. 1). Thus, on board processed data are less reliable than raw data to perform quantitative analysis^1,2,13,16^.

Resolving the controversy about the source of the basal reflections might require a cross-frequency analysis^17^. Thus far, MARSIS’s companion, the higher frequency (20 MHz) radar SHARAD (Shallow Radar), on board MRO, has been unable to probe through the SPLD down to the base of large portions of the south polar cap. Therefore, SHARAD observations cannot presently be used to impose additional constraints on the properties of the material at the base of the ice. Importantly, MARSIS acquires data at three different frequencies (3, 4, and 5 MHz) making the investigations of the frequency behavior of the signals propagating through the SPLD and reflected by the basal material possible. To date, though, frequency analysis has only been employed to verify the reliability of the data collected at Ultimi Scopuli^1^ but has yet to be employed to characterize the physical conditions in the subsurface.

Here, we analyze information from different MARSIS frequencies to compute the attenuation of the radar signal, to constrain the SPLD composition, and to evaluate the possible range of temperatures at the base of the ice. The results of this work provide new key information to narrow the range of interpretations on the nature of possible sources of the bright basal reflections detected in Ultimi Scopuli.

## Results

### Radar attenuation

Absorption of radar signals propagating through a medium causes reduction of the signal intensity (attenuation). Attenuation depends on the frequency of the traveling signal, the length of the path, and the type of material through which the signal propagates. This parameter can be computed by two different approaches, which are dictated by the radar performance and the subsurface properties. In RES studies, attenuation is usually estimated from the measurements of the intensity of the signal reflected by internal layering^18,19^. This method was tested on MARSIS^20^ and SHARAD^21^ data. Another technique measures the variation of echo power from a single subsurface interface observed at different depths^22–31^. Here we combine the latter method with a procedure introduced by ref. 32, based on MARSIS data acquired at the three operating frequencies (3, 4, and 5 MHz).

**Fig. 1:**
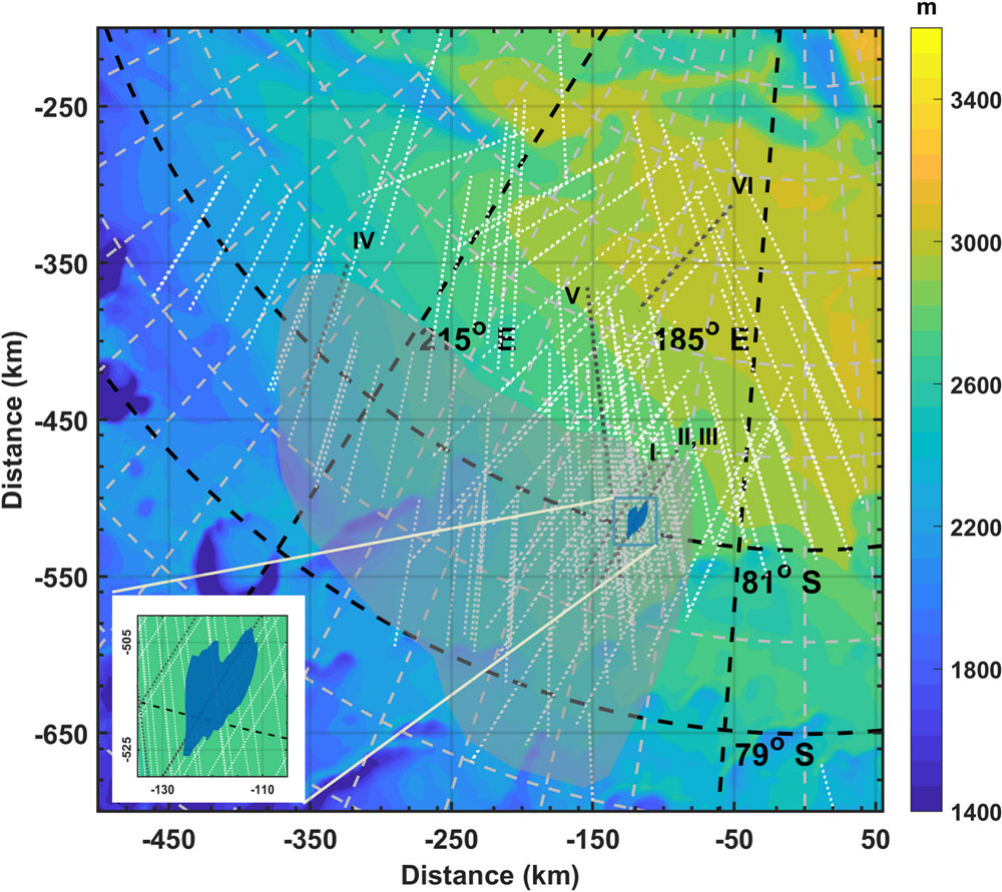
Mars Orbiter Laser Altimeter (MOLA) topographic map of the investigated area at Ultimi Scopuli. Dotted lines are MARSIS observations. The blue region indicates the geographic location of the main bright area. The observations in the light-gray shadowed area have not been used for data inversion, as they cross high and low basal reflectivity areas and cannot be assigned neither to bright nor to non-bright datasets. Black dotted lines refer to observations plotted in Fig. 2: 2654(V), 10737(II), 12685(III), 12780(VI), 14967(I), 19392(IV). Two tracks (II and III) across the central part of the main bright area, are shown by the same black dotted line on the map. The map is created using MATLAB software and MOLA data.

**Fig. 2:**
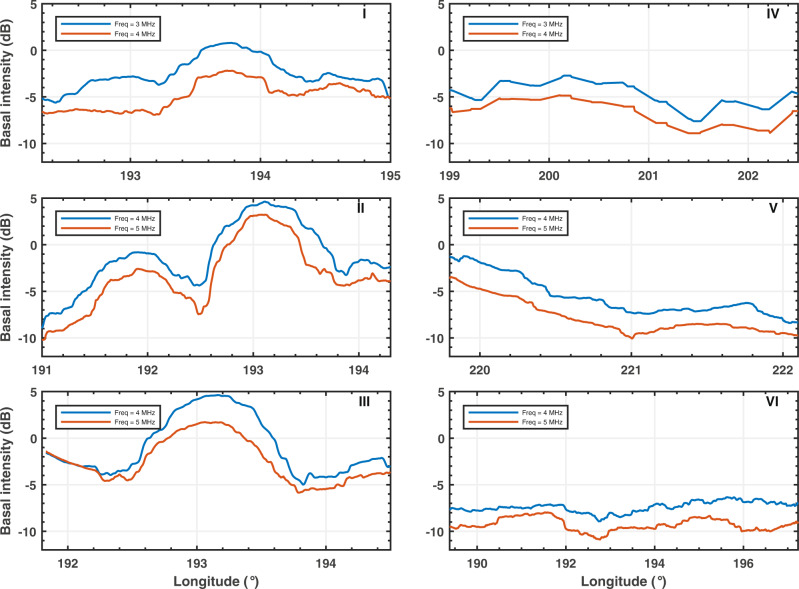
Basal normalized echo power measured at two frequencies. The plots refer to observations collected very close (I) or inside (II, III) and outside the main bright area (IV, V, VI) of Fig. 1, after applying an along track average^2^. Note that plots II and III do not totally overlap and start/end at different locations. The difference in basal power between frequencies is approximately constant along track and does not appreciably change between observations, aside from a small segment in observation III.

Along the same track, MARSIS simultaneously collects radar data as 3 MHz and 4 MHz pairs, or 4 MHz and 5 MHz pairs, generating two radar profiles for each observation (i.e., each radar track). The dataset used in the present analysis consists of 132 MARSIS raw observations, acquired at Ultimi Scopuli between 2010 and 2019 (Fig. 1): 36 at 3 MHz, 132 at 4 MHz and 96 at 5 MHz. Such observations have been collected on a large region, were both bright and non-bright areas were detected^2^. Regardless of the location (inside or outside the bright areas), the echoes collected at the higher frequency have smaller amplitude than those collected at the lower frequency (Fig. 2). This frequency-dependent behavior can be due to the properties of the SPLD, which affect the propagation of the signals, and/or to the properties of the basal interface, which control the intensity of the reflection; the latter depends on the interface roughness and the dielectric contrast between the SPLD and the underlying material (see Methods). The contribution of the interface roughness can be evaluated through the computation of the signal acuity, defined as a parameter that measures the smoothness of the interface^2^. We found that the calculated acuity values are uncorrelated (correlation coefficient <0.1) to the values of the difference in echo power between two frequencies: this implies that the roughness effect on the frequency behavior of the reflected signal is actually negligible. Furthermore, because the difference in basal echo power at two frequencies is constant both within and outside the bright areas (Fig. 2), we can also exclude a frequency effect due to the basal dielectric contrast. We thus conclude that the frequency behavior of MARSIS data is primarily controlled by the properties of the SPLD.

**Fig. 3:**
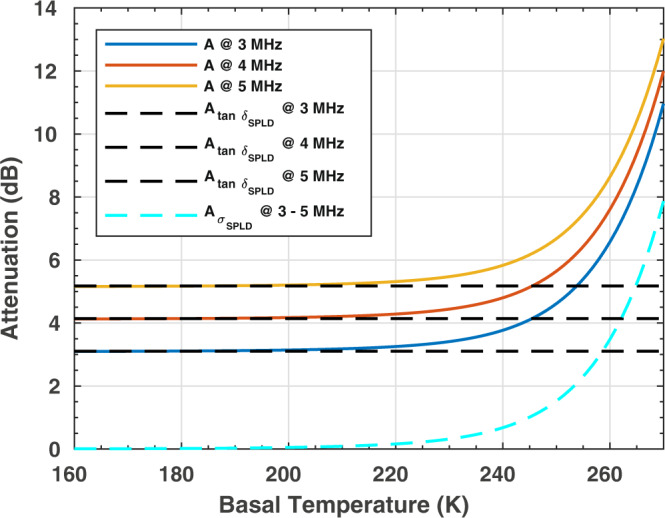
Attenuation in an ice/dust mixture at MARSIS frequencies. Solid lines are computed using Maxwell-Garnett mixing formula (see Methods) assuming a $12\%$ dust content and ${{\tan }}{\delta }_{d}=1.7\times{10}^{-2}$, admixed with pure water ice. Black dashed lines are computed using the same mixing formula and negligible ice conductivity. The cyan dashed line is computed assuming negligible dust loss tangent. All curves refer to $17.5\,{{\upmu {{{\rm{s}}}}}}$ two-way travel time.

At the MARSIS operating frequencies, the signal attenuation in water ice is frequency independent^33^, unless the ice contains impurities^34^, such as mineral inclusions, in which case attenuation of the signal in the mixture reflects the frequency-dependent behavior of the impurities (solid lines in Fig. 3). Vice versa, while attenuation in pure water ice is temperature dependent, presence of dust modifies such behavior, so that attenuation of the radar signal in the mixture becomes temperature independent at low temperatures. Attenuation, A, in the ice/dust mixture composing the SPLD is given by two contributions:

1 $$A={A}_{{\sigma }_{{{{{{\rm{SPLD}}}}}}}}{A}_{{{\tan }}{\delta }_{{{{{{\rm{SPLD}}}}}}}}={e}^{-\frac{{\sigma }_{{{{{{\rm{SPLD}}}}}}}}{{\varepsilon }_{0}{\varepsilon }^{{\prime} }}\tau }{e}^{-2\pi \nu \,{{\tan }}\,{\delta }_{{{{{{\rm{SPLD}}}}}}}\tau }.$$

where ${\varepsilon }_{0}=8.85\times 1{0}^{-12}\,{{{{{\rm{F}}}}}}/{{{{{\rm{m}}}}}}$ is the dielectric permittivity in a vacuum, ${\varepsilon }^{{\prime} }$ is the real part of permittivity of the SPLD, $\nu$ is the frequency, and τ is the two-way travel time (see Methods). The first exponential gives the attenuation of the conductivity of the SPLD (${A}_{{\sigma }_{{{{{{\rm{SPLD}}}}}}}}$) (cyan dashed line in Fig. 3); it is temperature dependent and frequency independent. The second exponential gives the attenuation due to the polarization phenomena and it is expressed by the loss tangent of the mixture (${A}_{{{\tan }}{\delta }_{{{{{{\rm{SPLD}}}}}}}}$) (Black dashed lines in Fig. 3); it is temperature independent and frequency dependent.

### Loss tangent and dust estimation

**Fig. 4:**
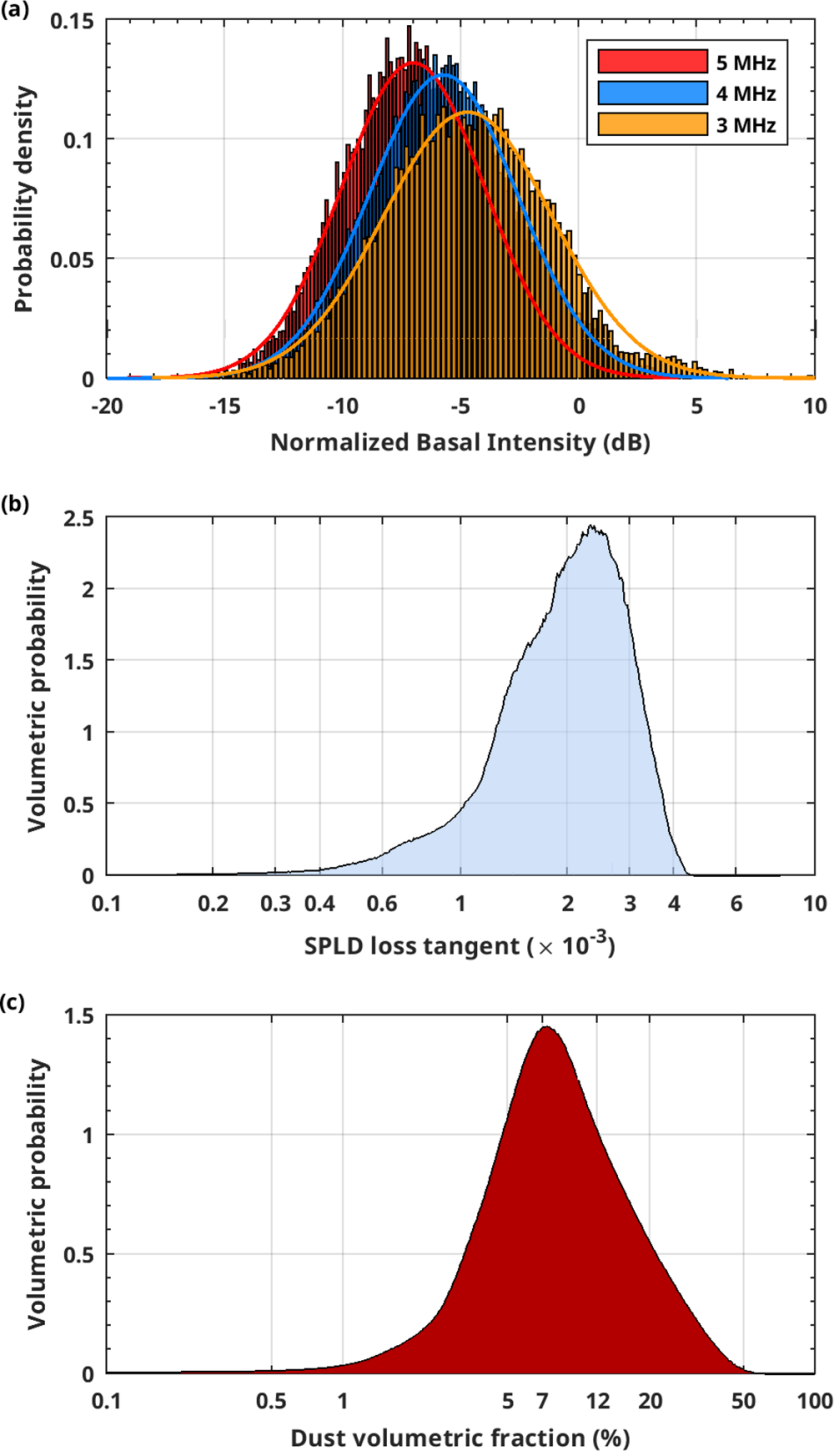
Analysis of MARSIS data collected outside the bright areas. **a** Normalized basal power distributions at three frequencies measured for different two-way travel times, in the range $13{-}25\,{{{{{\rm{\mu }}}}}}{{{{{\rm{s}}}}}}$; **b** Volumetric probability of the estimated SPLD loss tangent, from which the 25th percentile (${{\tan }}{\delta }_{{{{{{\rm{SPLD}}}}}}}=1.5{{{{\times}}}}{10}^{-3}$) and 75th percentile (${{\tan }}{\delta }_{{{{{{\rm{SPLD}}}}}}}=2.6{{{{\times}}}}{10}^{-3}$) have been calculated; **c** dust volumetric probability from which the range $5{-}12\%$, corresponding to same percentiles, has been estimated.

The best conditions to invert Eq. (1) and retrieve the loss tangent, are realized outside of the shadowed area (Fig. 1): here the two-way travel time τ of the basal reflector varies between $13\,{{{{{\rm{\mu }}}}}}{{{{{\rm{s}}}}}}$ and $25\,{{{{{\rm{\mu }}}}}}{{{{{\rm{s}}}}}}$ and the number of samples is large at all frequencies (Fig. 4a); moreover, we can assume that the dielectric contrast between the SPLD and the basal material is relatively constant. This assumption is supported by the fact that dry materials have comparable dielectric permittivities. The calculated ${{\tan }}{\delta }_{{{{{{\rm{SPLD}}}}}}}$ values range between $1.5 \times {10}^{-3}$ (25th percentile) and $2.6 \times {10}^{-3}$ (75th percentile), with the highest probability density corresponding to a loss tangent value $2.4\times {10}^{-3}$ (Fig. 4b). In the dataset collected in the bright areas (gray shadowed area in Fig. 1) the basal reflectivity changes abruptly along track (Fig. 2), as discussed in detail in Lauro et al.^2^, and the two-way travel time τ remains essentially constant. In these conditions it is not possible to apply the method described above, however the subset of data collected in the main bright area (blue region in Fig. 1), where the basal reflectivity is quite constant, can still be used to estimate the loss tangent from the difference in echo power at two frequencies^32^. We found values of the same order of magnitude to those estimated outside the bright areas but distributed within a larger range ($1.6 \times {10}^{-3}$ to $5.8 \times {10}^{-3}$). Moreover, we detected a very peculiar radar feature inside the main bright area (Supplementary Fig. 2): a strong multiple reflection visible in several MARSIS observations. In terrestrial radar data strong multiple reflections are caused by very large dielectric contrasts, such as those between ice and liquid water or brines^35^. These features cannot be produced by dielectric contrasts of smaller magnitude, for example at the interface between water ice and frozen soil or solid rocks^36,37^. The analysis of such multiple provides similar loss tangent values as those computed outside and inside the main bright area (Supplementary Fig. 2).

Assuming a dust loss tangent ranging between $7 \times {10}^{-3}{{{{{\rm{and}}}}}}\;4 \times {10}^{-2}$ which are typical values for terrestrial and lunar basalts and shergottite^38,39^, we thus estimate that the amount of dust in the water ice is between 5% (25th percentile) and 12% (75th percentile; Fig. 4c) (see Methods). In our calculations we did not consider any fraction of CO_2_ ice in the SPLD which, even if present, would have no effect on our estimate of the dust content, owing to the fact that the CO_2_ ice loss tangent is in the order of 10^−3^ (Supplementary Fig. 3).

### Basal permittivity estimation

**Fig. 5:**
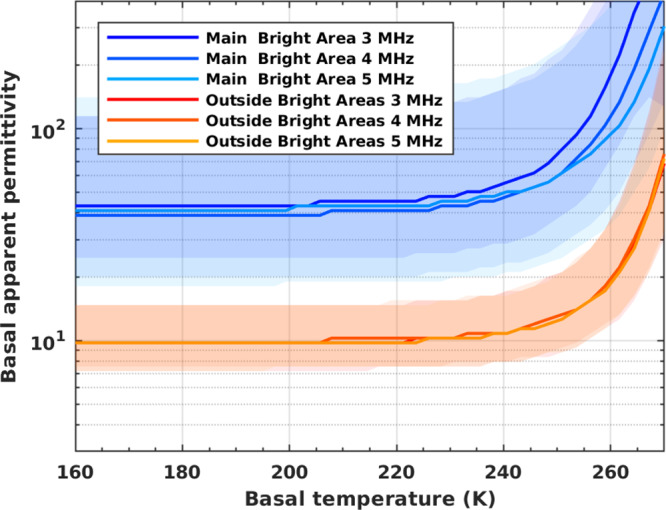
Apparent basal permittivity inside and outside the bright area as a function of basal temperature. Solid color lines are the median values at three frequencies, and the color bands indicate the $25{{{{{\rm{th}}}}}}{-}75{{{{{\rm{th}}}}}}$ percentile range of apparent permittivity values. Above $230\,{{{{{\rm{K}}}}}}$ the median values estimated for both areas increase towards very high values.

The loss tangent values estimated here are larger than that previously recognized^1,2^ implying a higher attenuation in the SPLD, which requires new calculations of the values of apparent basal permittivity. In earlier work^1^, these were frequency dependent, with medians of 30, 33, 22 inside the bright area and 9.5, 7.5, 6.7 outside the bright area, at 3, 4 and 5 MHz, respectively. To calculate the revised values of apparent basal permittivity, we applied the inversion procedure published by ref. 12, filtering the data for an acuity value >0.6 to mitigate the effect of the roughness of the base, consistently with what we discussed in previous work^2^. Here, we report the results obtained considering different thermal scenarios for the SPLD and the basal material, assuming a linear temperature increase inside the SPLD, from a fixed surface temperature ($160\,{{{{{\rm{K}}}}}}$) to a variable value at the base of the ice ($160{-}270\,{{{{{\rm{K}}}}}}$) (Fig. 5). The permittivity values are generally constant at temperatures between $160$ and $\sim 210\,{{{{{\rm{K}}}}}}$, step slightly upward beyond $210\,{{{{{\rm{K}}}}}}$, and increase abruptly at a ${{{{{\rm{T}}}}}}\ge 230\,{{{{{\rm{K}}}}}}$. Outside the bright areas, the apparent permittivity values range from 7 (25th percentile) to 12 (75th percentile) with a median of 10, regardless of frequency. Inside the main bright area (blue region in Fig. 1), the median of the distribution is $\sim 40$ at all frequencies, with values ranging between 20 (25th percentile) and 120 (75th percentile). The attenuation computed here appears reliable because it corrects the apparent permittivity values computed at three frequencies and makes those values consistent, as they should be once the frequency effect is accounted for. This correction works well both inside and outside the bright areas, and it is evidence that the SPLD are compositionally and thermally homogeneous in Ultimi Scopuli. Finally, we note that in both areas, the trend of the apparent permittivity with temperature, diverges at values larger than $230\,{{{{{\rm{K}}}}}}$. Above such temperature the attenuation of the SPLD becomes progressively larger (Fig. 3) thus a higher basal apparent permittivity is required to produce the same intensity of the echo. Such high value, estimated outside the bright areas, appears to be unreliable when compared to those typical of dry and cold rocks. We thus conclude that the temperatures of the SPLD at Ultimi Scopuli cannot exceed $230\,{{{{{\rm{K}}}}}}$, which can then be taken as the upper limit value of basal temperatures.

### Thermal profile of the SPLD and basal temperature

We calculated the temperature at the base of the SPLD applying the one-dimensional Fourier’s law of heat conduction ${{{{{\boldsymbol{q}}}}}}=-\!k\nabla T$ with ${{{{{\boldsymbol{q}}}}}}$ the heat flux at the base of the ice deposits, $k$ the thermal conductivity of the deposits, and $\nabla T$ the temperature gradient (see Methods). We must also know the temperature at the top of the deposits. None of these parameters have been directly measured yet, and their estimated values are model dependent. Published^1,7,10^ values for local surface temperatures are $155\,{{{{{\rm{K}}}}}}$, $\sim 160\,{{{{{\rm{K}}}}}}$ and $162\,{{{{{\rm{K}}}}}}$ (derived from the models by ref. 40), to which we add an estimated value of $\sim 170\,{{{{{\rm{K}}}}}}$ obtained averaging the summer and winter temperatures calculated from the thermophysical properties of water ice at these latitudes^41^. The local heat flux at the planet surface varies from $\sim 22\,{{{{{\rm{mW}}}}}}/{{{{{{\rm{m}}}}}}}^{2}$ to $\sim 28\pm 5\,{{{{{\rm{mW}}}}}}/{{{{{{\rm{m}}}}}}}^{2}$ based on geochemical criteria^42,43^ (i.e., abundance of radioactive elements in the crust and mantle, and bulk planetary composition), while radar, gravity and topography data applied to a flexural loading model^11^ suggest values $< 23.5\,{{{{{\rm{mW}}}}}}/{{{{{{\rm{m}}}}}}}^{2}$. It is however worth noting that recent data from the InSight mission suggest that the Martian upper crust may contain a much higher concentration of radioactive elements than previously thought^44^, and therefore models of global surface heat flow might need to be revised upward.

The thermal conductivity of the SPLD is the least well constrained parameter, depending strongly on the nature and abundance of the materials that form the bulk of the deposits, namely water ice and dust. The thermal conductivity of Martian dust is based on measurements of terrestrial simulants consisting of fine-grained materials (${{{{\phi }}}}:1{-}3\,{{{{{\rm{\mu }}}}}}{{{{{\rm{m}}}}}}$) developed by various laboratories^45,46^. A new value of thermal conductivity for Martian dust of $0.015\,{{{{{\rm{W}}}}}}/{{{{{\rm{mK}}}}}}$ has been recently reported for a temperature of $243\,{{{{{\rm{K}}}}}}$ and pressure equal to $650\,{{{{{\rm{Pa}}}}}}$, based on laboratory measurements conducted on a newly developed simulant (Jining Martian Dust Simulant: JMDS-1), that very closely resembles both the mineralogy and grain size of typical Martian dust^47^, unlike other simulants used thus far.

The temperature dependence of the thermal conductivity of water ice is commonly described by^48^ ${{{{{\rm{k}}}}}}=12.52-6.90\times {10}^{-2}{{{{{\rm{T}}}}}}+1.15\times {10}^{-4}{{{{{{\rm{T}}}}}}}^{2}$, or by a simplified formulation^49^, as: ${{{{{\rm{k}}}}}}=567/{{{{{\rm{T}}}}}}$, with ${{{{{\rm{T}}}}}}$ being the temperature expressed as K. Because of the limited quantity of dust in the deposits, we assumed that dust is uniformly distributed in the ice and calculated the thermal conductivity at 1 m increments along the depth of the SPLD, using constant weighted averages (see Methods). We used the thermal conductivity obtained for each step to calculate the value of temperature at that depth and used this value to calculate the thermal conductivity for the next step, terminating the iterations when a depth of $1.5$ km was reached. The range of values of basal temperatures we obtained for varying combinations of dust/ice ratio in the range 5–12% dust (based on our estimates), heat flux and surface temperature, varies between a minimum of $168\,{{{{{\rm{K}}}}}}$ and a maximum of $186\,{{{{{\rm{K}}}}}}$ (Table 1: Sets 1 and 2, respectively).

**Table 1:** SPLD physical parameters for basal temperature computation

Parameter	Set 1	Set 2
Heat flux $q$ (mW/m^2^)	22	30
$Z$: Thickness of SPLD (m)	1500	1500
$\varDelta z$: depth increment (m)	1	1
${T}_{0}$ (top temperature) (K)	160	170
${k}_{{{{{{\rm{dust}}}}}}}$ (W/mK)	0.015	0.015
${k}_{{{{{{\rm{water\; ice}}}}}}}$ (W/mK)	4.424 (at ${T}_{0}=\,160\,{{{{{\rm{K}}}}}}$)	4.1135 (at ${T}_{0}=170\,{{{{{\rm{K}}}}}}$)
Fraction of dust (%)	5	12
${T}_{b}$ (basal temperature) (K)	168	183 (186)*
Corrected for density (${{{{{\rm{K}}}}}}$)	176	193

*Calculated using Klinger^49^.

The parameter values we used to calculate the basal temperatures from the heat conduction equation are fully consistent with geological interpretations and data obtained from decades of observations and measurements by a variety of instruments. Here, we show that a modest change in the parameter values returns broadly different values of basal temperature, without the need to advocate for special conditions (e.g., an anomalously high heat flux, or different SPLD materials).

If we consider the effect of the porosity of the ice, which works toward decreasing the value of thermal conductivity of the SPLD, we can expect higher temperatures at the base of the ice. Even when compacted at high pressure, the residual porosity of water ice remains high (e.g., 0.1 at 150 MPa^50^). Additionally, inhomogeneities in the ice matrix, such as those that accommodate dust or other impurities, contribute to the brittle behavior of the ice sheet, favoring the formation of fractures, and increasing its relative volume of voids. In the absence of robust data on the porosity of the SPLD across their vertical extent, we used the density of pure ice as a proxy, knowing that it varies with temperature according to the equation^51^ *ρ* = 917 ⋅ [1−1.17 ⋅ 10^−4^ *θ*], applicable within the temperature (${{{{{\rm{\theta }}}}}}$) interval from 0 °C (273 K) to −140 °C (133 K). When we account for the variation of density along the thermal profile of the SPLD, we can determine its effect on the variability of the thermal conductivity of the pure ice component, using the equation^52^ $k=3.176\times {10}^{-3}{\rho }_{i}-0.726$ where the density of ice ${\rho }_{i}$is expressed as ${{{{{\rm{kg}}}}}}/{{{{{{\rm{m}}}}}}}^{3}$. This equation was obtained by linear least-square fit (correlation coefficient = $0.996$) from experiments conducted on a broad range of water ice densities ($620{-}915\,{{{{{\rm{kg}}}}}}/{{{{{{\rm{m}}}}}}}^{3}$), which we extrapolated to the density values we calculated for the SPLD ($925 {-}929$ ${{{{{\rm{kg}}}}}}/{{{{{{\rm{m}}}}}}}^{3}$). For the same set of conditions shown in Table 1, the basal temperatures range from a minimum value of $176\,{{{{{\rm{K}}}}}}$ (Set 1, Table 1) to a maximum of $193\,{{{{{\rm{K}}}}}}$ (Set 2, Table 1).

The temperatures thus obtained could be even higher if we included a thermally insulating surficial layer of dust, as done elsewhere^7^. Furthermore, in our computations of the content of dust, we did not account for possible CO_2_ ice in the SPLD. Because the presence of CO_2_ ice would not appreciably change the estimated amount of dust (Supplementary Fig. 3), even if we were to consider CO_2_ ice layers in our calculations, the total proportion of water ice would be reduced, which would lead to further reduction of the thermal conductivity of the deposits, with consequent further increase of the calculated basal temperature values. We thus conclude that the values of basal temperatures so far published in some of the literature, very likely underestimate the temperature at the base of the SPLD, and that a temperature $\sim 200\,{{{{{\rm{K}}}}}}$ (consistent with $205\,{{{{{\rm{K}}}}}}$ reported by ref. 1) represents a reasonable assumption to further discuss the physical properties of the SPLD.

## Discussion

**Fig. 6:**
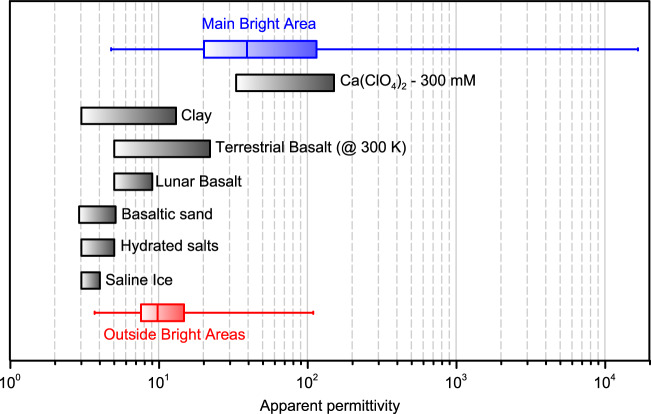
Apparent permittivity of various materials compared to those retrieved by MARSIS at Ultimi Scopuli. The box plots indicate the basal apparent permittivity retrieved inside the main bright area (blue) and outside the bright areas (red), where the whiskers represent the minimum and maximum values, the box extremes the lower and upper quartiles, and the center line the median of the data distributions. The gray bars indicate the range of apparent permittivity values for proposed basal materials measured at MARSIS operating frequencies^3,34,38,39,53–67^: calcium perchlorate at $196{-}200\,{{{{{\rm{K}}}}}}$ (Supplementary Fig. 5); clays at $200\,{{{{{\rm{K}}}}}}$; terrestrial basalts at $300\,{{{{{\rm{K}}}}}}$; lunar basalts at $200\,{{{{{\rm{K}}}}}}$; hydrated salts jarosite and meridianite at $200\,{{{{{\rm{K}}}}}}$; saline ices at $200\,{{{{{\rm{K}}}}}}$-; and basaltic sand at $200\,{{{{{\rm{K}}}}}}$.

The attenuation estimated in this work shifted upward the apparent permittivity values (Fig. 5) retrieved by MARSIS data inversion with respect to previous calculations^1^. Inside the main bright area, the median of the apparent permittivity distribution is 40 and outside is 10, regardless the frequency (3, 4, and 5 MHz). Moreover, in the range $$160{-}210\,{{{{{\rm{K}}}}}}$$, these values are constant. To identify potential candidates as SPLD basal materials, we compared the apparent permittivity values retrieved at $$200\,{{{{{\rm{K}}}}}}$$ with those computed from literature data (Fig. 6) relevant to subglacial lithologies suggested in recent papers^1–6^. Each material is represented by a range of values which reflects the variability of data present in the literature, aside from 300 mM Ca(ClO_4_)_2_ brines which are new data (Supplementary Fig. 5). Where possible, we reported values collected around $$200\,{{{{{\rm{K}}}}}}$$ however, terrestrial basalts were mostly measured at higher temperatures (around $$300\,{{{{{\rm{K}}}}}}$$), often with poorly controlled moisture content^53^, and must be considered an overestimation of the corresponding values for Martian temperatures^54^. In fact, in agreement with dielectric behavior of rocks, if such values were measured at lower temperatures ($$200\,{{{{{\rm{K}}}}}}$$) the entire range would shift leftward towards smaller apparent permittivity values^53^, $$ < 15$$. The comparison clearly highlights that 75% of the apparent permittivity distribution outside the bright areas is compatible with materials having apparent permittivity lower than 15, like terrestrial/lunar basaltic rocks or clay sediments, at Martian temperature. Conversely, 75% of the apparent permittivity distribution inside the main bright area is only compatible with the 300 mM Ca(ClO_4_)_2_ brines, which have reported values of apparent permittivity larger than 20.

Previously published basal temperatures (e.g., Sori and Bramson^7^) fall below the temperatures required for brines to be liquid. In this paper, however, we have shown that it is possible to calculate basal temperatures much closer to the eutectic temperatures of perchlorate brines ($\sim 198\,{{{{{\rm{K}}}}}}$), if a broader spectrum of environmental conditions of dust and ice (fully consistent with the evidence acquired over decades of Martian observations) are considered. It is therefore not implausible to envisage specific mechanisms that might further raise basal temperatures beyond those of the brine eutectics, so long as the basal temperature does not exceed our attenuation-derived modeled maximum basal temperature of $230\,{{{{{\rm{K}}}}}}$. While speculative at this stage, we hypothesize that salt could be enhanced in the basal unit of the SPLD via significant sublimation of a paleo SPLD that formed and was lost during large obliquity variations. Additionally, salts could have been concentrated in a depositional environment, such as a paleolake that then dried up during large obliquity variations. Additional raw MARSIS data over a larger extent of the SPLD will help determine if the reflectors in Ultimi Scopuli are unique or part of a larger collection of bright reflectors^14^.

As an aside, our estimation of the attenuation in the SPLD suggests a plausible explanation as to the reason why SHARAD has thus far not been able to penetrate through the full depth of the deposits to detect the basement. Considering a loss tangent of the order of $1.5{-}2.6 \times {10}^{-3}$, and neglecting the conductive term due to the low temperature at the base of the SPLD, Eq. (1) can be used to compute the attenuation at SHARAD frequency (20 MHz), which ranges between $-14$ and $-25\,{{{{{\rm{dB}}}}}}$. Given such values and the dynamic range of SHARAD, the presence of any additional scattering loss due to the structure of the SPLD may prevent the propagation of the signal at large depth, thus explaining why SHARAD cannot “see” the bright reflectors at Ultimi Scopuli and, more generally, the basal interface below the SPLD.

## Methods

### Ice dielectric properties

Loss tangent is the fundamental parameter retrieved in this work to estimate the SPLD dust content and signal attenuation. Such parameter is given by two contributions that behave differently vs. temperature and frequency. The dielectric response to an oscillating electric field (e.g., a radar wave) is described by the complex relative dielectric permittivity of the medium:

2 $$\varepsilon={\varepsilon }^{{\prime} }-i\left({\varepsilon }_{p}^{{\prime} {\prime} }+\frac{\sigma }{\omega {\varepsilon }_{0}}\right)={\varepsilon }^{{\prime} }(1-i \,{{\tan }}\delta )$$

where ${\varepsilon }_{0}=8.85\times 1{0}^{-12}\,F/m$ is the dielectric permittivity in a vacuum, $\sigma$ is the conductivity, $\omega=2\pi \nu$ is the angular frequency, and $\nu$ the frequency. The real part of the dielectric permittivity, ${\varepsilon }^{{\prime}}$, accounts for the polarizability of the material (energy storage) and the imaginary part for the energy loss due to heat caused by the polarization  process (${\varepsilon }_{p}^{{\prime} {\prime} }$) and free charge carrier migration (conduction, $\sigma$). It is common to define in Eq. (2) the total losses through the loss tangent factor ${{\tan }}\delta$ which is the sum of the conductive term ${{\tan }}{\delta }_{\sigma }=\sigma / \omega {\varepsilon }_{0}{\varepsilon }^{{\prime} }$ and the polarization term ${{\tan }}{\delta }_{p}={\varepsilon }_{p}^{{\prime} {\prime} }/{\varepsilon }^{{\prime} }$:

3 $${{\tan }}\delta={{\tan }}{\delta }_{\sigma }+{{\tan }}{\delta }_{p}$$

In water ice admixed dust, at MARSIS frequencies, ${{\tan }}{\delta }_{\sigma }$ strongly depends on temperature (${\propto e}^{-\frac{{E}_{a}}{{kT}}}$) and decreases with frequency ($\propto 1/ \nu$), whereas ${{\tan }}{\delta }_{p}$ is both frequency and temperature independent.

### Radar equation

The relation between loss tangent and basal power measured by MARSIS describes the forward model used in this work. According to radar equation in case of a normally impinging wave propagating in a medium delimited by two interfaces (air/SPLD and SPLD/basal material), the ratio between basal and surface echo intensities (${P}_{b}\,{{{{{\rm{and}}}}}}\,{P}_{s}$) can be written as^55^:

4 $$\frac{{P}_{b}}{{P}_{s}}=\frac{{{{{{{\rm{\chi }}}}}}}_{{{{{{\rm{b}}}}}}}}{{\chi }_{s}}{\left[\frac{\left(1-{{\rho }_{s}}^{2}\right){\rho }_{b}}{{\rho }_{s}}\right]}^{2}A,$$

where ${{{{{\rm{\chi }}}}}}$ accounts for interface roughness, $\rho$ is the Fresnel coefficient, the suffixes *b* and *s* refer to basal and surface quantities, respectively and *A* represents the attenuation factor given by:

5 $$A\simeq {e}^{-2{\int }_{0}^{H}\omega \frac{\sqrt{{\varepsilon }^{{\prime} }}}{c}\,{{\tan }}\, \delta \left(z\right){dz}}={e}^{-2{\int }_{0}^{H}\frac{\sigma \left(z\right)\sqrt{{\varepsilon }^{{\prime} }}}{{\varepsilon }_{0}c}{dz}-2{\int }_{0}^{{{{{{\rm{H}}}}}}}\omega \frac{\sqrt{{\varepsilon }^{{\prime} }}}{c}{{\tan }}\,{\delta }_{p}\left(z\right){dz}}={e}^{-\frac{{\sigma }_{{{{{{\rm{SPLD}}}}}}}}{{\varepsilon }_{0}{\varepsilon }^{{\prime} }}\tau -\omega {{\tan }}\,{\delta }_{{{{{{\rm{SPLD}}}}}}}\tau },$$

where $z$ is the depth, $H$ is the SPLD thickness, $\tau=\frac{2H\sqrt{{\varepsilon }^{{\prime} }}}{c}$ is the two-way travel time, ${{\tan }}{\delta }_{{{{{{\rm{SPLD}}}}}}}$ and ${\sigma }_{{{{{{\rm{SPLD}}}}}}}$ are the overall SPLD loss tangent and conductivity, respectively.

Selecting only MARSIS data with high acuity $( > 0.6)$ the roughness terms can be neglected: ${{{{{{\rm{\chi }}}}}}}_{{{{{{\rm{b}}}}}}}/{\chi }_{s}\simeq 1$; in addition, as the temperature expected for the SPLD is very low, conductivity losses are negligible (Supplementary Fig. 4). Under these assumptions, Eq. (4), expressed in logarithmic scale, results linearly dependent on frequency $\nu$ and time delay $\tau$^34^:

6 $${\hat{P}}_{n}\left(\nu,\,\tau \right)\simeq {\hat{P}}_{{{{{{\rm{n}}}}}}0}-\xi \nu \;{{\tan }}\;{\delta }_{{{{{{\rm{SPLD}}}}}}}\tau,$$

where $${\hat{P}}_{n}\left(\nu,\tau \right)=10\,{{{\log }}}_{10}\left(\frac{{P}_{b}}{{P}_{s}}\right)$$, $$\xi=2\pi \,10{{{\log }}}_{10}e$$, and $${\hat{P}}_{{{{{{\rm{n}}}}}}0}= 10 \,{{{\log }}}_{10} \!\! {\left(\frac{\left(1-{{\rho }_{s}}^{2}\right){\rho }_{b}}{{\rho }_{s}}\right)}^{2}$$.

### Loss tangent estimation

Equation (6) was used to estimate the loss tangent using a probabilistic approach^56,57^, defined by the forward model ${\hat{P}}_{n}=g(m)$ (Eq. 6) and the model parameters $m=({\hat{P}}_{{{{{{\rm{n}}}}}}0},{{{\log }}}_{10}({{\tan }}{\delta }_{{{{{{\rm{SPLD}}}}}}}))$. The estimation was carried out considering the data collected outside the bright areas (Fig. 1), where the time delay $\tau$ varies between 13 and 25 $\upmu$s; the dataset ${\hat{P}}_{n}$ was divided in 21 subsets (7 time delay intervals for each frequency) having 2$\,{{{{{\rm{\mu }}}}}}$s width. For each subset (${\nu }_{i},{\tau }_{j}$) the probability distribution ${p}_{{{{{{\rm{ij}}}}}}}$ of ${\hat{P}}_{n}\left({\nu }_{i},{\tau }_{j}\right)$ was retrieved and the posterior volumetric probability ${\sigma }_{M}(m)$ computed as the product of the probability calculated for each subset ${p}_{{{{{{\rm{ij}}}}}}}$:

7 $${\sigma }_{M}(m)={p}_{M}(m){\prod }_{{{{{{\rm{ij}}}}}}}\sqrt{\frac{\det ({g}_{m}+{D}^{t}{g}_{d}D)}{\det ({g}_{m})}}{p}_{{{{{{\rm{ij}}}}}}}({\hat{P}}_{n}=g(m)),$$

where ${g}_{m}$ is the metric of the model parameters space, ${g}_{d}$ the metric of the data space, ${D}_{{kl}}=\partial {g}_{k}/\partial {m}_{l}$ and ${p}_{M}\left(m\right)$ the prior probability of the model parameters.

The loss tangent marginal volumetric probability was computed as

8 $$p\left({{\tan }}{\delta }_{{{{{{\rm{SPLD}}}}}}}\right)=\int {\sigma }_{M}\left(m\right)d{\hat{P}}_{{{{{{\rm{n}}}}}}0},$$

considering that the prior probability on ${\hat{P}}_{{{{{{\rm{n}}}}}}0}$ is described by a uniform distribution: $p({\hat{P}}_{{{{{{\rm{n}}}}}}0})={const},\, {\hat{P}}_{{{{{{\rm{n}}}}}}0}\in [-4,\, 1]$ dB; the range of ${\hat{P}}_{{{{{{\rm{n}}}}}}0}$ was computed assuming a typical rock as basal material (permittivity values ${\varepsilon }_{b}\in [8,\, 12]$) and water ice admixed with dust for the SPLD (${\varepsilon }_{{{{{{\rm{SPLD}}}}}}}\in [3.1,\, 3.5]$).

### Dust volume fraction estimation

In an icy mixture, loss tangent value is strongly affected by the dust content and can be considered a reliable parameter to estimate the amount of dust. Given the water ice/dust composition of the SPLD, the overall complex permittivity ${\varepsilon }_{{{{{{\rm{SPLD}}}}}}}$ can be written using Maxwell-Garnett formula^58^:

9 $${\varepsilon }_{{{{{{\rm{SPLD}}}}}}}={\varepsilon }_{{{{{{\rm{ice}}}}}}}+3{f}_{v}{\varepsilon }_{{{{{{\rm{ice}}}}}}}\frac{{\varepsilon }_{d}-{\varepsilon }_{{{{{{\rm{ice}}}}}}}}{{{\varepsilon }_{d}+2{\varepsilon }_{{{{{{\rm{ice}}}}}}}-f}_{v}\left({\varepsilon }_{d}-{\varepsilon }_{{{{{{\rm{ice}}}}}}}\right)},$$

where ${\varepsilon }_{{{{{{\rm{ice}}}}}}}$ is the complex permittivity of pure water ice at 200 K^59^, ${\varepsilon }_{d}=8.8 \times (1-i \,{{\tan }}{\delta }_{d})$ is the complex permittivity of the dust and, ${f}_{v}$the dust volumetric fraction. The estimation of ${f}_{v}$ is based on a probabilistic approach where the data are described by the estimated volumetric probability $p({{\tan }}{\delta }_{{{{{{\rm{SPLD}}}}}}}),$the physical model by $g\left(m\right){\mathfrak{=}}{\mathfrak{I}}m\{{\varepsilon }_{{{{{{\rm{SPLD}}}}}}}\}{\mathfrak{/}}{\mathfrak{R}}e\{{\varepsilon }_{{{{{{\rm{SPLD}}}}}}}\}$(Eq. 9) and model parameters are $m=({{{\log }}}_{10}({f}_{v}),{{{\log }}}_{10}({{\tan }}{\delta }_{g}))$. The posterior probability is computed as,

10 $${\sigma }_{M}\left(m\right)={p}_{M}\left(m\right)\sqrt{\frac{{{\det }}\left({g}_{m}+{D}^{t}{g}_{d}D\right)}{{{\det }}({g}_{m})}}p({{\tan }}{\delta }_{{{{{{\rm{SPLD}}}}}}}=g \left(m\right)).$$

Given the paucity of dielectric properties measurements on Mars analogue solid samples, we considered loss tangent values of solid lunar samples available in the literature^39^ which allow us to establish a lower and upper limit in the dust loss tangent values. In particular, we considered a uniform distribution for the prior probability of the dust loss tangent $p\left({{\tan }}{\delta }_{d}\right)={const},\, {{\tan }}{\delta }_{d}\in [7 \times {10}^{-3},4 \times {10}^{-2}]$. We note that this range also includes the theoretical value $({{\tan }}{\delta }_{d}=2 \times {10}^{-2})$ of shergottite according to Olhoeft and Strangway^38^. It follows that the posterior volumetric probability of ${f}_{v}$ is given by

11 $$p\left({f}_{v}\right)=\int {\sigma }_{M}\left(m\right){dtan}{\delta }_{d}$$

### Thermal profile and basal temperature

The variation of temperature from the top to the base of the SPLD in Ultimi Scopuli is treated as a one-dimensional problem. We assume: (a) that the SPLD is a half space having uniform thickness $Z$, bounded by two horizontal planar surfaces, ${S}_{0}$ (top) and ${S}_{n}$ (base); (b) that the SPLD is homogeneous throughout, and no internal heat source exists; and (c) a steady-state scenario, for which Laplace equation ${\nabla }^{2}T=0$ is satisfied. Under these conditions, the heat flux ${q}_{n}$ at the base and at the top ${q}_{0}$ of the SPLD is the same, and constant throughout. For each columnar element of height Z and basal area $1\,{m}^{2}$, the condition: ${q}_{n}={q}_{0}={q}_{i}$ is satisfied, with ${q}_{n}$ and ${q}_{0}$ the heat flow through the basal surface (${s}_{n}$) and the top (${s}_{0}$) surface of the columnar element, respectively, and ${q}_{i}$ the heat flow for any generic horizontal surface element (${s}_{i}$) in between. The thermal profile from the top to the base is thus controlled by the temperature at the top (${T}_{0}$), the planetary heat flow in the region (*q*), and the thermophysical characteristics of the SPLD material. From Fourier’s law we have:

12 $$q=-\!k\frac{{dT}}{{dz}},$$

where $\frac{{dT}}{{dz}}$ is the temperature gradient, and $k$ is the thermal conductivity of the material, which depends on temperature, and therefore varies with depth. Considering a volume subdivided into $n-1$ discrete elements of equal height $\triangle z$, we rewrite Eq. (12) as

13 $$q=-\!{k}_{i}\frac{\triangle {T}_{i}}{\triangle z}$$

where $\triangle {T}_{i}={T}_{i}-{T}_{i+1}$ is the temperature difference between the top (${T}_{i}$) and bottom (${T}_{i+1}$) of the *i-th* volume element, and ${k}_{i}$ is considered constant inside the volume element, imposing as a starting condition: $k({T}_{1})=k({T}_{0})$ and therefore at the base: $k\left({T}_{n}\right)=k({T}_{n-1})$. Using this notation, we can describe the variation of temperature and corresponding variation of $k$ with depth as a simple problem of heat refraction: we assume that each elemental volume of height $\triangle z$ is a material with specific thermophysical characteristics (${k}_{i},\, {i}={{{{\mathrm{1,\, 2}}}}}\ldots n$) that are distinct and different from those of the elemental volumes above and below. Therefore:

14 $$q=-\!{k}_{1}\frac{{\triangle T}_{1}}{{\triangle z}_{1}}=-\!{k}_{2}\frac{{\triangle T}_{2}}{{\triangle z}_{2}}=\ldots=-\!{k}_{n}\frac{{\triangle T}_{n}}{{\triangle z}_{n}}$$

We thus use a finite difference method to describe the variation of temperature with depth in the SPLD to calculate the basal temperature ${T}_{n}$, using appropriate $k\propto 1/T$ functions.

## Supplementary information

Supplementary Information

## Data Availability

Data used in this study are available in the Zenodo database https://zenodo.org/record/3948005.
