# Supplementary material for: Using MARSIS signal attenuation to assess the presence of South Polar Layered Deposit subglacial brines
Source: Nat Commun. 2022 Sep 28;13:5686. doi: 10.1038/s41467-022-33389-4 (PMC9519933; doi:10.1038/s41467-022-33389-4)
Supplement: Supplementary file 1 — Supplementary Information [file 41467_2022_33389_MOESM1_ESM.pdf]

Supplementary information

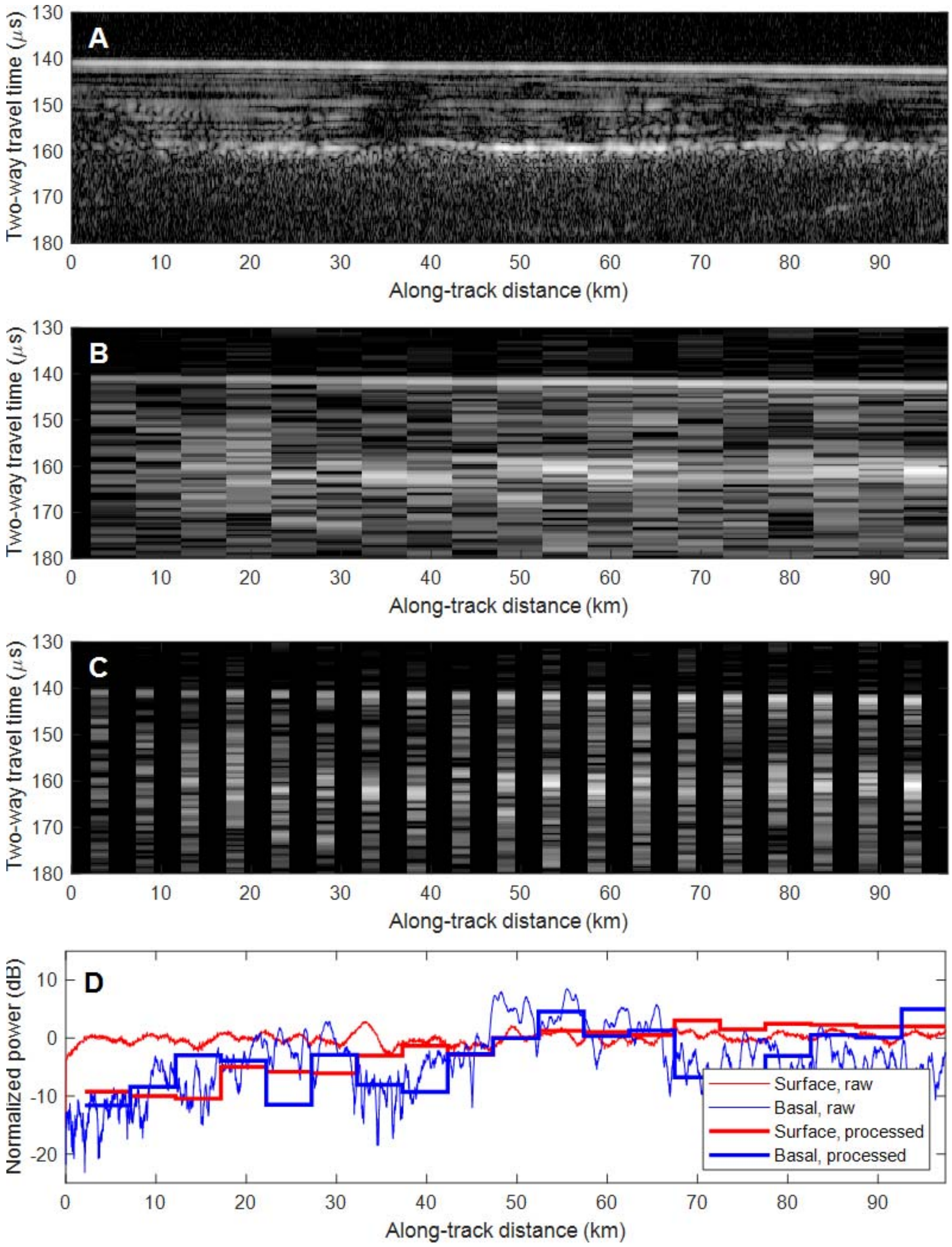

Supplementary Figure 1 - Simulation of the effect of on-board processing in MARSIS echoes. (A) Radargram for MARSIS orbit 10737, produced using the same data utilized in Figure 2[1]. (B) Radargram computed from the same data shown in (A), after application of the on-board processing algorithms[2]. On-board processing

consists of taking a group of echoes, compensating for the relative shift in echo delay time due to the vertical motion of the spacecraft, and summing them together to improve along-track resolution and signal-to-noise ratio. The exact number of echoes is computed through a formula found in[2] and depending on altitude, horizontal velocity and wavelength. Processed echoes are shown to extend over the along-track distance corresponding to the time over which the original raw echoes were acquired and processed. (C) The same radargram in (B) is shown with only the actual echoes used in on-board processing. Thus, processed echoes are shown to extend over the along-track distance corresponding to the time over which the original raw echoes were acquired. Gaps in along-track coverage larger than actual data are revealed by this representation. (D) Comparison between surface and subsurface echo power in raw and on-board processed data. Data from each radargram are normalized to the median of the corresponding surface echo power values, for ease of comparison. Subsurface power in processed data does not exhibit the same peaks visible in raw data, presumably because of sampling and averaging effects in on-board coherent summation.

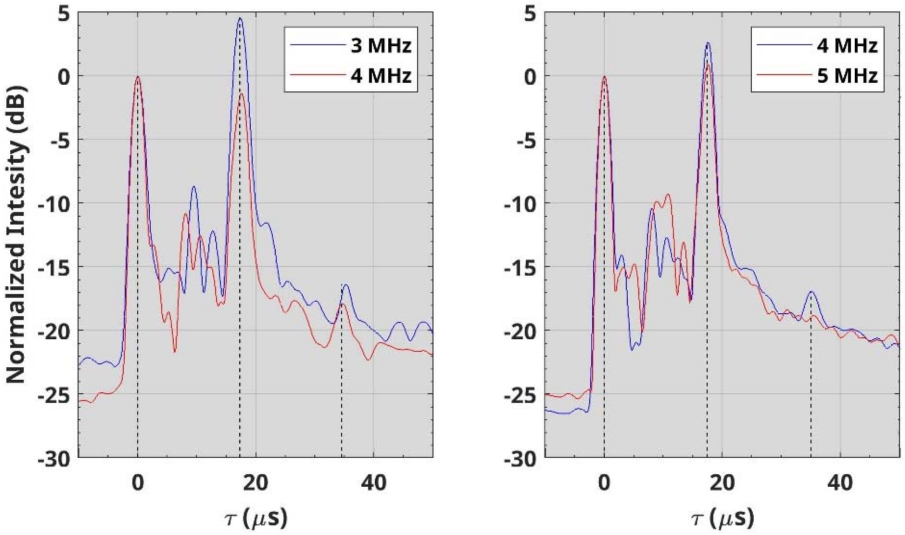

Supplementary Figure 2. Plots of MARSIS traces collected inside the main bright area. The radar traces are extracted from two observations (15110 and 10737) collected at 3 and 4MHz (a) and 4 and 5 MHz (b). The

multiple reflections are clearly visible at a time depth of about 35μs, which is double the two-way travel time of the basal reflections (17.5 μs). Such multiple reflections have been used to compute the loss tangent from the difference in echo power at two frequencies[3]. The estimated values range between $1x10^{-3}$ and $4x10^{-3}$.

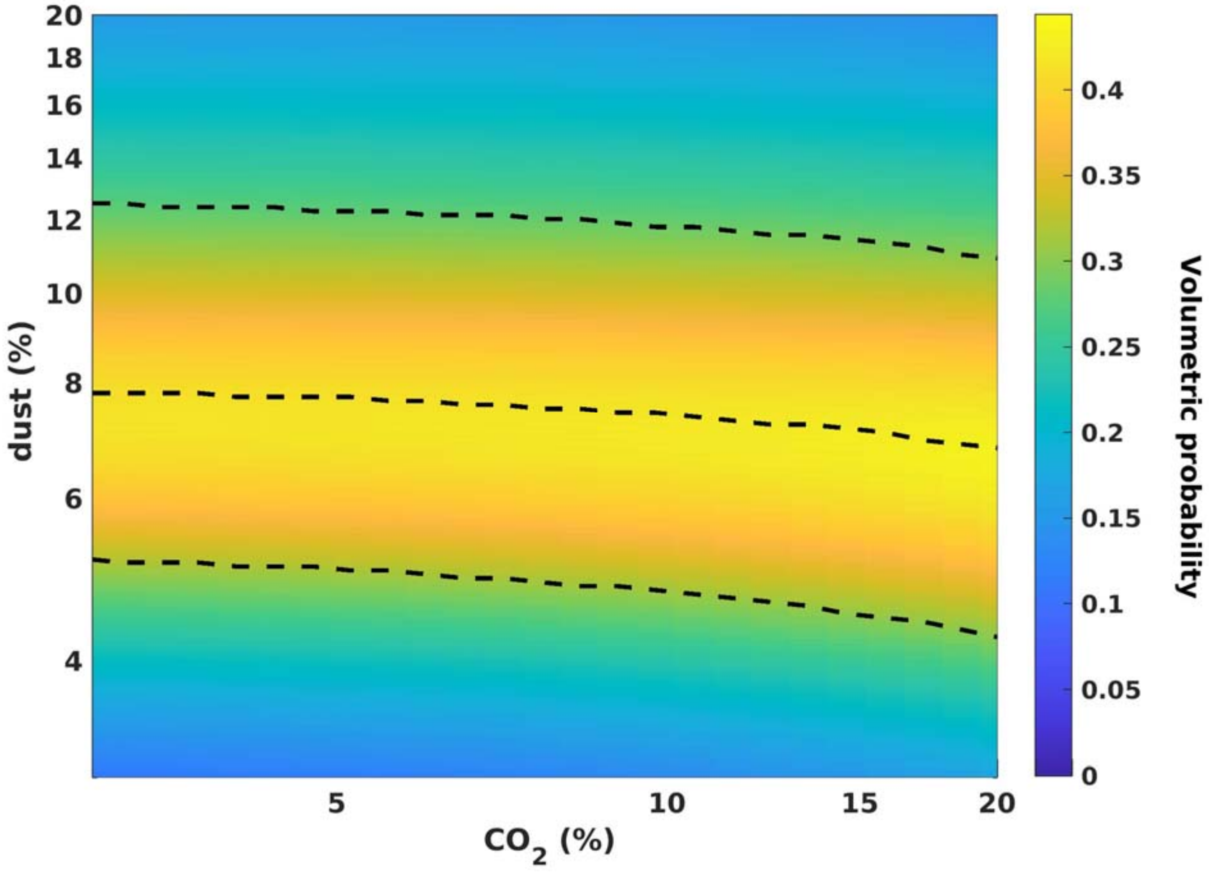

Supplementary Figure 3. Effect of CO2 ice on the estimation of the dust content in the SPLD. Estimation of dust content for a three-phase system H_2O ice – CO_2 ice - mineral dust where $\varepsilon_{CO_2} = 2.2 \times (1 - i10^{-3})$ (see Methods). The image represents the volumetric probability where dashed black lines indicate the range of dust as a function of the CO_2 content, for 25th, 50th and 75th percentiles. Note that the amount of dust only weakly depends on the CO_2 content.

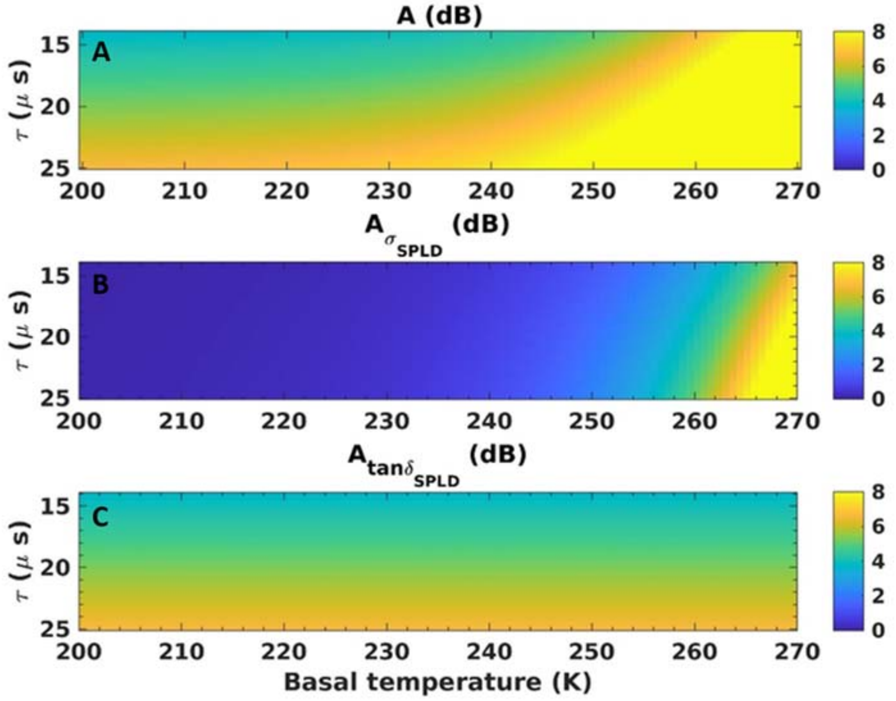

Supplementary Figure 4. Effect of conductivity and dust loss tangent on the signal attenuation in the SPLD. Attenuation computed at 4 MHz as function of the two-way travel time and basal temperature. (A) SPLD attenuation computed using Maxwell-Garnett mixing formula (see Methods), admixing pure water ice with 12% dust having $tan\delta_d = 1.7x10^{-2}$. (B) SPLD attenuation computed as in (a) neglecting the dust loss tangent and (C) neglecting the conductivity of water ice.

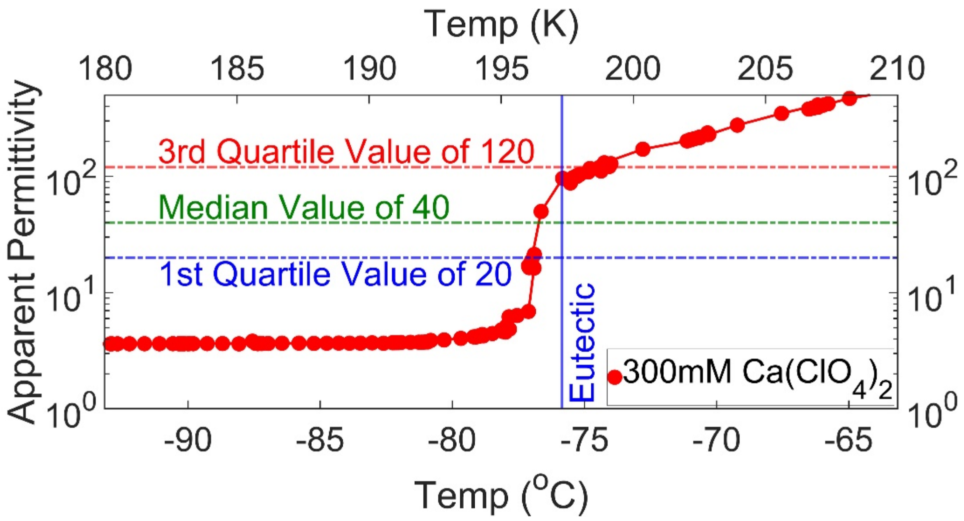

Supplementary Figure 5. Temperature dependence of the apparent permittivity of a 300 mM (7wt%) $Ca(ClO_4)_2$ solution. Apparent permittivity drastically increases with increasing temperature as the liquid vein networks thaw at the eutectic temperature. The parabolic behavior of the apparent permittivity just before the eutectic temperature is reached due to premelting[4]. Measurement was made in a similar fashion to those previously described in Grimm et al.[4].

Supplementary References

1. Orosei, R. et al. Radar evidence of subglacial liquid water on Mars. Science 361, 490-493 (2018).
2. A. Cicchetti et al. Observations of Phobos by the Mars Express radar MARSIS: Description of the detection techniques and preliminary results. Adv. in Space Res. 60, 2289-2302 (2017).
3. Mattei, E. et al. Dielectric measurements and radar attenuation estimation of ice/basalt sand mixtures as martian Polar Caps analogues. Icarus 229, 428-433 (2014).
4. Grimm, R. E., Stillman, D. E., Dec, S. F., and Bullock, M. A. Low-frequency electrical properties of polycrystalline saline ice and salt hydrates. J. Phys. Chem. B 112, 15382-15390 (2008).
